# Discontent in Mental Hospitals

**Published:** 1913-12-20

**Authors:** F. J. Stuart

**Affiliations:** Provisional Secretary, Association of Assistant Medical Officers. Berry Wood, Northampton


### THE EDITOR'S LETTER-BOX.

### Discontent in Mental Hospitals.

*To the Editor of* The Hospital.

Sir,—I thank you for having devoted so much space to the question of the formation of an Association of Assistant Medical Officers of Asylums. Your report, however, of my remarks at London does not convey quite the same meaning as I wished. In stating that an assistant medical officer was married and had to live apart from his wife, I did not desire that it should be supposed that that was a common practice; I was merely giving an example of what I consider the worst disability that a man could suffer under. I hope it is an isolated case; it is a state of affairs that public opinion would not tolerate for one second, and in speaking to assistant medical officers I pointed out that asylum committees are elected bodies, and as such are subject to public opinion. An Association of Assistant Medical Officers has many ways of interesting asylum committees in the grievances of its members without intervening in triennial elections.—Yours faithfully,

F. J. Stuart, Provisional Secretary,
Association of Assistant Medical Officers.

Berry Wood, Northampton,
December 14.

